# Oleuropein Aglycone Protects Transgenic *C. elegans* Strains Expressing Aβ42 by Reducing Plaque Load and Motor Deficit

**DOI:** 10.1371/journal.pone.0058893

**Published:** 2013-03-08

**Authors:** Luisa Diomede, Stefania Rigacci, Margherita Romeo, Massimo Stefani, Mario Salmona

**Affiliations:** 1 Department of Molecular Biochemistry and Pharmacology, Istituto di Ricerche Farmacologiche “Mario Negri”, Milan, Italy; 2 Department of Biomedical Experimental and Clinical Sciences, University of Florence, Florence, Italy; 3 Research Centre on the Molecular Basis of Neurodegeneration (CIMN), Florence, Italy; 4 National Institute of Biostructures and Biosystems (INBB), Rome, Italy; Thomas Jefferson University, United States of America

## Abstract

The presence of amyloid aggregates of the 42 amino acid peptide of amyloid beta (Aβ42) in the brain is the characteristic feature of Alzheimer’s disease (AD). Amyloid beta (Aβ deposition is also found in muscle fibers of individuals affected by inclusion body myositis (sIBM), a rare muscular degenerative disease affecting people over 50. Both conditions are presently lacking an effective therapeutic treatment. There is increasing evidence to suggest that natural polyphenols may prevent the formation of toxic amyloid aggregates; this applies also to oleuropein aglycone (OLE), the most abundant polyphenol in extra virgin olive oil, previously shown to hinder amylin and Aβ aggregation. Here we evaluated the ability of OLE to interfere with Aβ proteotoxicity *in vivo* by using the transgenic CL2006 and CL4176 strains of *Caenorhabditis elegans*, simplified models of AD and of sIBM, which express human Aβ in the cytoplasm of body wall muscle cells. OLE-fed CL2006 worms displayed reduced Aβ plaque deposition, less abundant toxic Aβ oligomers, remarkably decreased paralysis and increased lifespan with respect to untreated animals. A protective effect was also observed in CL4176 worms but only when OLE was administered before the induction of the Aβ transgene expression. These effects were specific, dose-related, and not mediated by the known polyphenolic anti-oxidant activity, suggesting that, in this model organism, OLE interferes with the Aβ aggregation skipping the appearance of toxic species, as already shown *in vitro* for Aβ42.

## Introduction

Alzheimer’s disease (AD) is the most common form of dementia, affecting a large proportion of aged people in the developed countries, where its social impact and burden on national health budgets are severe [1]. The key histopathological sign of AD is the presence, in several brain areas, of intracellular neurofibrillary tangles of hyperphosphorylated tau and of minute extracellular amyloid deposits (plaques) around cerebral vessels and dystrophic and degenerating neurites [2], [3]. The main component of the plaques is a polymeric fibrillar form of one out of a family of 39–43 amino acid variants (particularly the 42 amino acid peptide, Aβ42) of a peptide generated by proteolysis of the membrane amyloid precursor protein (AβPP) [4]. The presently accepted “amyloid cascade hypothesis” states that the functional alterations and behavioral deficits that characterize AD result primarily from the presence of these plaque deposits [5]. More recently, the interest in deciphering the relation between plaque burden, tissue functional impairment and neuronal death has focused on the importance, as the main toxic species to neurons, of the oligomeric pre-fibrillar assemblies originating at the onset of peptide polymerization into fibrils [6]–[10]. It has also been proposed that intraneuronal Aβ may play a crucial role in the early progression of AD [11]; accordingly, the focus of the research into molecules able to delay AD occurrence and to relieve its symptoms has shifted from hindering fibril growth to avoiding the appearance of toxic oligomeric intermediates.

Aβ deposits and oligomers are also found in inclusion body myositis (sIBM), a musculoskeletal pathology which, while rare, is the most frequent degenerative condition progressively affecting muscular apparatus in patients over 50. The pathology displays a progressive muscle weakness and atrophy resulting in severe disability [12]. Biopsy analysis of the diseased tissue shows vacuolated muscle fibers containing deposits of ubiquitin-positive aggregates of misfolded proteins, including Aβ and phosphorylated tau, which display amyloid signatures, together with remarkable inflammation, similar to AD and other neurodegenerative pathologies [13], [14]. The importance of Aβ levels for disease development and progression of muscle degeneration was also supported by a study on transgenic mice engineered so as to produce increased amounts of Aβ42 in the muscle tissue [15].

In spite of the intense efforts of the international research aimed at unraveling the pathophysiology of cell degeneration and tissue functional impairment, AD and sIBM still remain diseases without an effective therapy. Nevertheless, many molecules have been investigated as possible drugs useful in relieving AD occurrence and symptoms [16], [17], and some of them entered clinical trials. At present, the search of therapeutically exploitable molecules also focuses on dietary regimens epidemiologically associated with reduced risk of developing AD or with significant delay in the appearance of AD symptoms in the aged population. Mounting evidence supports the idea that the Mediterranean diet (MD), rich in polyphenols, is greatly beneficial for the prevention of age-related dysfunctions as well as of several diseases, including cancer and cardiovascular events, cerebrovascular disease, stroke and neurodegenerative diseases [18]–[21], notably vascular dementia and AD. Studies in rodents have found that foods rich in polyphenols, particularly those present in wine and in extra virgin olive oil (EVOO), improve deficits in learning and memory associated with aging and disease, reverse oxidative damage in the brain [22], [23] and attenuate AD-like pathology and cognitive deterioration in the Tg2576 AD mouse model 24.

Natural phenolic substances, a vast array of molecules found in many plants and foods of vegetal origin, can hinder amyloid aggregation in several ways; most of them (epigallocatechin gallate, oleocanthal, curcumin, oleuropein aglycone (OLE), resveratrol, ellagic acid, tannic acid) display the ability to prevent the appearance of those pre-fibrillar aggregates considered the most toxic amongst amyloid species both in cultured cells [25]–[31] and in animal models [32]–[34]. A number of studies, including the “Three city study” [35] have clearly shown a strict association between most of the protective effects of the MD and the sustained consumption of EVOO, a basic component of the MD. In particular, a number of polyphenols and secoiridoids found in EVOO, including oleocanthal, hydroxythyrosol and OLE, have been considered potential candidates as key responsible of the protective effect of EVOO [30], [31], [36], [37]. Oleuropein (the glycoside of OLE) is a main constituent of the leaves and unprocessed olive drupes of *Olea europaea*, and most of the phenolic molecules found in olive oil or table olives result from spontaneous oleuropein hydrolysis and processing. Oleuropein hydrolysis is carried out by an endogenous β-glycosidase during olive ripening and in the technological process of olive oil production and releases the aglycone moiety of the molecule in the oil [38]. The finding that oleuropein associates with the Aß peptide into a non-covalent complex [36], [39] supports our recent data showing that OLE interferes with the *in vitro* aggregation of human amylin and Aβ42, skipping the appearance of toxic oligomers and promoting peptide aggregation into aggregates devoid of cytotoxicity [30], [40].

Here we report an *in vivo* study on OLE protection against Aβ42 aggregation in tissue, which generates the plaque deposits found in AD and in sIBM, using *C. elegans* as a simplified invertebrate model of AD [41], [42] and, possibly, of sIBM. In particular, we used the CL2006 transgenic *C. elegans* strain, constitutively expressing cytoplasmic human Aβ_3–42_ in the body wall muscle cells [41], [42]. This strain displays an age-related progressive reduction of muscle-specific motility which is related to the accumulation of both Aβ_3–42_ fibrils and oligomers [17], for this reason, it has already been used to demonstrate the protective effect of several treatments, including the *Gingko biloba* extract EGb 761 and tetracyclines, against Aβ toxicity *in vivo* [43]–[48]. We show that, unlike CL2006 worms grown on normal medium, those grown on OLE-supplemented medium are protected against plaque deposits, Aβ oligomer appearance and the resulting impairment of motility, as well as displaying increased survival.

Our results support the possibility that dietary supplementation with OLE can be beneficial against sIBM and AD by reducing plaque burden as well as by delaying the occurrence and reducing the severity of symptoms.

## Materials and Methods

### Materials

Tetracycline hydrochloride was from Fluka (Switzerland) and n-acetyl-cysteine (NAC) was from Sigma Aldrich (Switzerland). All drugs were freshly dissolved in water before use. Oleuropein was from Extrasynthese (Lyon, France). Almond β-glycosidase (EC3.2.1.21) was from Fluka (Sigma-Aldrich, Steinheim, Germany). Oleuropein deglycosylation by β-glycosidase was performed as described [40]. The complete deglycosylation was confirmed by assaying the glucose released in the supernatant with the Glucose (HK) Assay Kit (Sigma). GC-MS (gas cromatography-mass spectrometry) analysis showed the absence of any oleuropein in the precipitate and the substantially total recover of OLE in the same precipitate. A 100 mM stock solution of OLE was made in dimethyl sulfoxide (DMSO); dilutions in aqueous buffers were made immediately before use.

### C. elegans strains

The N2 ancestral strain, the transgenic CL2006 strain, the temperature-inducible transgenic nematode strain CL4176 and the control CL802 strain were used [41], [42]. CL2006 constitutively produces Aβ_3–42_ in the body-wall muscles, while the expression of muscle-specific Aβ_1–42_ in CL4176 depends on raising the temperature from 16 to 24 °C. The transgenic arrays in the CL2006, CL4176 and CL802 strains all contain the dominant mutant collagen [rol-6 (su 1066)] as morphological marker. All nematode strains were obtained from the Caenorhabditis Genetic Center (University of Minnesota, USA) and were propagated at 16 °C on solid Nematode Growth Medium (NGM) seeded with *E. coli* (OP50) for food. To prepare age-synchronized animals, the nematodes were transferred to fresh NGM plates on reaching maturity at 3 days of age and allowed to lay eggs overnight. Isolated hatchlings from the synchronized eggs (day 1) were cultured on fresh NGM plates at 16 °C.

### Paralysis assay

The N2 wild type strain, and the CL2006 and CL802 worms, after egg synchronization, were placed at 16 °C on fresh NGM plates (35×10 mm culture plates, 100 worms/plate) seeded with OP50 *E. coli*. At L1, L2 or L3 larval stage, the worms were fed with different concentrations of OLE (12.5–500 µM, 100 µl/plate) and paralysis was evaluated at L4 larval stage. The worms that did not move or only moved their head when gently touched with a platinum loop were scored as paralyzed. Tetracycline was used as positive control. To this end, egg-synchronized CL2006 worms placed on tetracycline-resistant *E. coli* at 16 °C were fed, at L3 larval stage, with 50 µM tetracycline and the paralysis was scored after 24 h [48].

CL4176, after egg synchronization, were placed on fresh NGM plates (35×10 mm culture plates, 100 worms/plate) seeded with *E. coli* for 54 h at 16 °C. To induce the transgene expression, the temperature was raised from 16 to 24 °C. Worms were treated with vehicle or OLE (50–500 µM, 100 µl/plate) 30 h before (corresponding to L1 larval stage), 6 h before (corresponding to L2 larval stage) or 18 h after the temperature shift (corresponding to L3 larval stage) (Figure S1A). The effect of repeated administration of 50–100 µM OLE at L1, L2 and L3 was also determined. The paralysis was scored 42 h after the temperature induction. Tetracycline, at 50 µM, was administered 18 h after temperature rise, as positive control, and the paralysis was scored 24 h after treatment [48].

### Lifespan

Gravid CL2006 and CL802 worms were allowed to lay eggs for 6–8 h to produce an age-synchronized population which was cultured at 16 °C. At L2 larval stage, the worms were fed with vehicle or with 50–100 µM OLE. In some experiments, a second administration of OLE was performed after 8 days. Once the worms reached adulthood (at reproductive maturity), they were transferred daily to fresh NGM plates seeded with OP50, in the absence of fluorodeoxyuridine, until they stopped laying eggs. To avoid overlapping generations, the worms were then transferred every day until all nematodes were dead. The number of worms paralyzed (considered dead) was scored starting from 48 h after treatment, at L4 larval stage (day 0), and for each consecutive day until all worms were dead.

### Fluorescence staining of β-amyloid

Age-synchronized CL2006 worms, either fed or not fed with OLE as described above, were fixed in 4% paraformaldehyde in PBS, pH 7.4, for 24 h at 4 °C. The nematodes were stained with 1.0 mM 1,4-bis(3-carboxy-hydroxy-phenylethenyl)-benzene (X-34), in 10 mM Tris-HCl buffer, pH 8.0, a dye specific for amyloid aggregates, for 4 h at room temperature [48]. The samples were then destained, mounted on slides for microscopy and observed with an inverted fluorescence microscope (IX-71 Olympus); the images were acquired using a CDD camera. Amyloid deposits in the anterior area of worms fed with vehicle (n = 20) and OLE (n = 20) were quantified by counting the number of X-34 positive spots and were expressed as Aβ deposits/anterior area.

### Aβ expression and content of toxic oligomers in transgenic CL2006

Transgenic worms, fed with vehicle or with 50 µM OLE, were collected by washing the plates with M9 buffer; the suspension was transferred to tubes, centrifuged at 1100×g for 4 min and washed twice to eliminate the bacteria. The worm pellet was then resuspended in lysis buffer (5.0 mM NaCl, 5.0 mM EDTA, 1.0 mM dithiothreitol (DTT) and protease inhibitor mixture in 25 mM Tris/HCl buffer, pH 7.5) and homogenized using a TeSeE homogenizer (Bio-Rad) with acid-washed glass beads (Sigma) [48]. The proteins were then precipitated overnight with methanol (1:4/vol:vol ) at −20 °C, before being resuspended in loading buffer. Equal amounts (5–10 µg) were then spotted onto nitrocellulose membranes (Millipore). A 0.1% Ponceau Red solution (Sigma Aldrich) was used to stain the blotted membranes for total protein visualization. After blocking with PBS plus 0.1% (v/v) Tween 20, the membranes were incubated overnight either with an anti-Aβ mouse monoclonal antibody (Clone WO2, 1:1000, Millipore) or with the A11 mouse monoclonal antibody (1:1000, Biosource), which specifically recognizes toxic Aβ pre-fibrillar oligomers. Peroxidase-conjugated anti-mouse IgG (1:5000, Sigma) was used as the secondary antibody. The mean volumes of immunoreactive spots and of Red Ponceau dyed spots were analyzed using the Progenesis SameSpots software (Nonlinear Dynamics, UK). The data were expressed as the mean of the volume of the immunoreactive spot/volume of total Ponceau dyed proteins in the spot ±SD.

### Superoxide production

Age-synchronized CL2006 or CL802 worms were placed on NGM plates seeded with *E. coli* at 16 °C for 48 h and then fed, at L2 larval stage, with vehicle or with 50 µM OLE for 48 h (100 µl/plate). As positive controls, worms were placed at 16 °C for 72 h, and then fed with 50 µM tetracycline or with 5.0 mM NAC for 24 h. The worms were collected into 1.6 ml of 1.0% Tween 20 in PBS and subjected to sonication using the Sonopuls Ultrasonic homogenizer (Bandelin, Germany) to break up the outer cuticle. 50 nM phorbol myristate acetate (PMA) and 1.8 mM Nitro Blue Tetrazolium (NBT) (Sigma Aldrich) were then added to the suspension. After incubation at 37 °C for 30 min, sample absorbance was measured at 560 nm using the Infinite M200 multifunctional micro-plate reader (Tecan, Austria). The protein content was determined using a Bio-Rad Protein assay (Bio-Rad Laboratories GmbH, Munchen, Germany). Results are calculated as absorbance units/mg of protein and expressed as % of control, i.e. the percentage of superoxide produced with respect to vehicle treated CL2006 worms.

### Statistical analysis

The effects of vehicle and drug on cultured worms were compared by an independent Student’s t-test or One-way ANOVA test, and the IC_50_ was determined using Prism version 4.0 for Windows (GraphPad Software, CA, USA). A *p*<0.05 was considered statistically significant.

## Results

**Figure 1:**
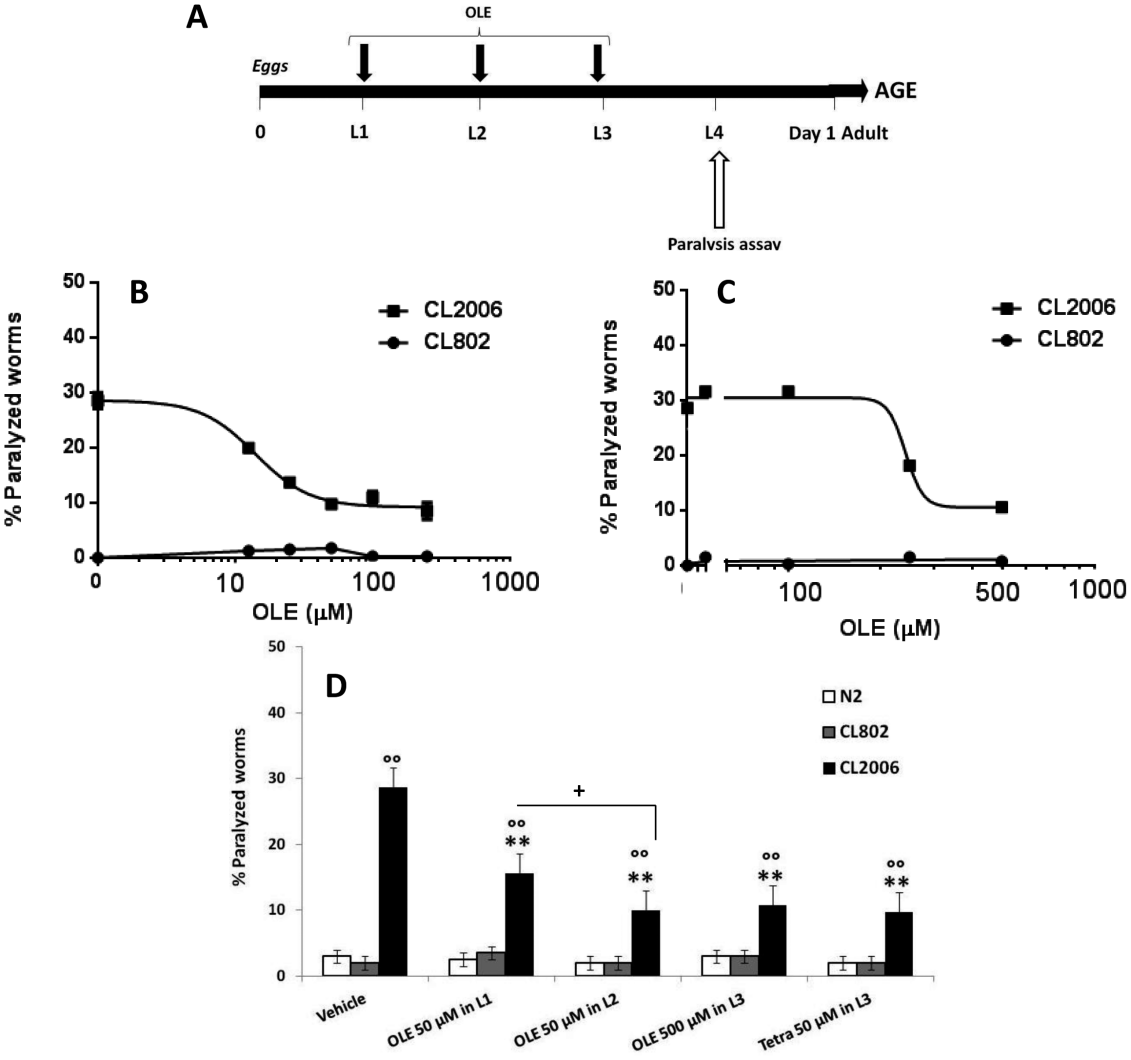
Effect of OLE on Aβ-induced paralysis in CL2006 transgenic *C. elegans* strain. (**A**) Diagram illustrating the paralysis assay showing when the drug was administered and when the paralysis assay was scored. (**B–C**) Dose-response effect of OLE on the paralysis induced by Aβ expression in CL2006 and CL802 transgenic worms. Egg-synchronized worms were placed at 16 °C on fresh NGM plates seeded with OP50 *E. coli* and, at L2 (**B**) or L3 (**C**) stage, were fed with OLE (12.5–500 µM). The number of paralyzed worms was scored 48 h or 24 h after treatment (at L4 larval stage) for L2- and L3-treated worms, respectively. Data are shown as percentage±SE of paralyzed worms to vehicle treated ones (n = 100 worms/group, 3 independent assays). (**D**) Percentage of paralyzed worms fed with OLE. CL2006, CL802 and N2 worms, cultured as above, were fed 50 µM OLE at L1 or L2 and 500 µM at L3. Tetracycline at 50 µM was administered at L3 as positive control. The number of paralyzed worms was scored at L4 larval stage. Data are shown as percentage±SD of paralyzed worms to vehicle treated ones (n = 100 worms/group, 3 independent assays). °° p<0.01 vs. CL802, **p< 0.01 vs CL2006 worms fed with vehicle (One-way ANOVA test), and +p<0.01 vs. CL2006 worms fed with 50 µM OLE at L2 (Student’s t test).

We have previously reported that OLE reduces Aβ aggregation *in vitro* and hinders the appearance of oligomeric pre-fibrillar aggregates, thus protecting the exposed cells against Aβ injury [30]. Here we checked whether OLE was also effective *in vivo* by investigating whether it protected the CL2006 strain against Aβ_3–42_ toxicity. The worms, synchronized and placed on *E. coli* at 16 °C, were fed with either vehicle or OLE (12.5–500 µM, 100 µl/plate), starting at L1, L2 or L3 larval stages. Worm paralysis was rated 96, 48 or 24 h later, when the worms were at L4 (see Fig. 1A for treatment schedule). N2 wild type strain and CL802 worms, in which the Aβ transgene was not expressed and the paralysis did not occur [43], were used as controls. We found that OLE protected CL2006 worms from the paralysis induced by Aβ expression and aggregation in a dose-dependent manner upon administration either at L2 or L3, supporting a specific effect induced by OLE (Fig.1B, C). The IC_50_ value, i.e. the drug concentration that inhibited paralysis by 50%, calculated after OLE administration at L2 larval stage, was 14.5±1.0 µM (n = 100 worms/group, mean±SE). This value was significantly lower (*p*<0.01, Student’s t test) than that determined after OLE administration at L3 (242±8.5 µM, n = 100 worms/group, mean±SE).

Feeding CL2006 worms at L1 larval stage with 50 µM OLE produced a protective effect significantly lower than that occurring when the same dose of drug was administered at L2 (45.8% vs. 65% reduction of paralysis for L1 and L2 administration, respectively, p<0.01, Student’s t test) (Fig. 1D) The protective effect at L1 did not increase by feeding worms with 100 µM OLE (46% protection of paralysis) and higher doses resulted to be toxic, causing the death of worms.

The protective effect of 50 µM OLE at L2 larval stage was similar to that occurring when 500 µM of drug was administered at L3; in fact, worm paralysis was reduced by 65.0% and 62.6% respectively (Fig.1D). The OLE effect was comparable to that obtained feeding CL2006 worms with 50 µM tetracycline at L3 (Fig. 1D), which resulted in a reduction of nematode paralysis of 66%. The effect of tetracycline when administered at L2 was not here considered due to the toxicity of the drug at this larval stage [48]. The double administration of 50 µM OLE both at L2 and L3 larval stage did not further improve drug protection; the reduction of the paralysis was comparable to that observed with the single administration at L2 (data not shown).

The feeding of CL802 worms and wild type N2 nematodes with OLE starting at L1 (50–100 µM), L2 or L3 larval stages (12.5–500 µM), did not modify their viability (Fig. 1B, C, D) indicating that the drug did not cause any aspecific and/or toxic effects in control strains.

We also investigated whether OLE protected CL4176 worms from the paralysis caused by the inducible Aβ_1–42_ expression [48]. According to the treatment schedule set out in Figure S1A, worms were fed either vehicle or OLE (50–500 µM, 100 µl/plate) 30 h before the temperature rise (at L1 larval stage), 6 h before the temperature rise (at L2) or 18 h after the rise of temperature (at L3). A slight reduction of the paralysis of CL4176 worms was observed only when nematodes fed 50–100 µM OLE 30 h before the induction of the Aβ expression (reduction of paralysis of 18%) (Fig. S1) whereas the other treatment schedules were ineffective (data not shown). Feeding CL4176 at L1 larval stage with OLE at doses higher than 100 µM, as well as the repeated administration of 50–100 µM OLE starting from L1, resulted to be toxic.

These findings indicated that the OLE protective effect was lower in CL4176 strain than in CL2006 worms: this could be ascribed to the fully penetrant paralysis phenotype of the inducible strain. The optimal protective effect of OLE against Aβ-induced paralysis was obtained in CL2006, when drug feeding started from L2 larval stage, and in CL4176 when the drug was administered from L1. These observations indicated that OLE protective effect was maximal when worms were treated from the beginning of the aggregation process.

**Figure 2:**
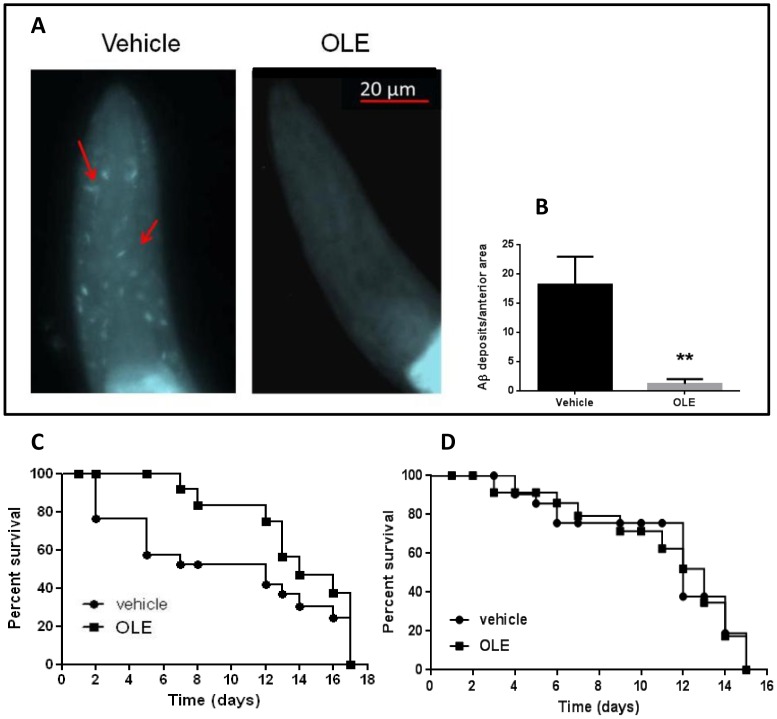
OLE reduces amyloid deposition and extends the life-span of CL2006 transgenic *C. elegans* strain. Egg-synchronized CL2006 worms were placed at 16 °C on fresh NGM plates seeded with OP50 *E.coli* and, at L2 larval stage, were fed with vehicle or with 50 µM OLE for 48 h. (**A**) At 120 h of age, corresponding to day 1 of adult age, worms were stained with X-34 dye. The staining of amyloid plaques on whole-mount and fixed samples was visualized at short wavelength excitation. Scale bar 20 µm. Arrows in red indicate amyloid deposits. (**B**) Amyloid deposits in the anterior area of worms fed with vehicle (n = 20) and OLE (n = 20), quantified by counting the number of X-34 positive spots. **p<0.01 vs. CL2006 worms fed with vehicle (Student’s t test). (**C–D**) Kaplan-Meier survival curves of (**C**) CL2006 and (**D**) CL802 worms fed with vehicle or with 50 µM OLE at L2 larval stage. The number of paralyzed worms (considered dead) was scored 48 h after treatment, at L4 larval stage (day 0 in graph) and every consecutive day, until all worms were dead. Survival is expressed as a percentage of the initial population. Plots are representative of three independent experiments (n = 30 worms/group).

We then investigated whether OLE reduced the degree of Aβ amyloidosis in CL2006 transgenic worms by evaluating the amount of aggregate deposits in their head region [49]. The worms, synchronized and placed at 16 °C for 48 h at L2, were fed either vehicle or OLE (50 µM, 100 µl/plate) for 48 h and then stained with the X-34 dye which recognizes β-amyloid aggregates [50]. OLE strongly reduced X-34 positive spots (Fig. 2A–B), indicating that the drug inhibited Aβ fibril deposition.

It is known that compounds able to interact with protein fibrils and aggregates affect age-related changes in protein homeostasis and extend worm lifespan [17]. Accordingly, we analyzed the effect of OLE administration on overall CL2006 and CL802 nematode survival. Feeding CL2006 worms at L2 larval stage with 50 µM OLE significantly increased their median survival by 47% (Fig. 2C) (median survival: 7 and 15 days for worms fed with vehicle or with OLE, respectively. *p* = 0.004, Log-rank test). The same effect on survival was observed after administration of a higher dose of OLE (100 µM) (median survival: 7 and 15 days for worms fed with vehicle or with OLE, respectively. *p* = 0.003, Log-rank test). In addition, the survival of CL2006 worms exposed to a double administration of 50 µM OLE, at L2 and 8 days after, was not different from that of nematodes receiving a single dose of the drug (median survival: 15 days for either worms fed one or two OLE doses. *p* = 0.790, Log-rank test).

The administration of 50 µM OLE at L2 larval stage to CL802 nematodes did not modify their survival (Fig. 2D) (median survival: 12 and 13 days for worms fed vehicle or OLE, respectively. *p*  = 1.000 Log-rank test) supporting the absence of aspecific effects.

**Figure 3:**
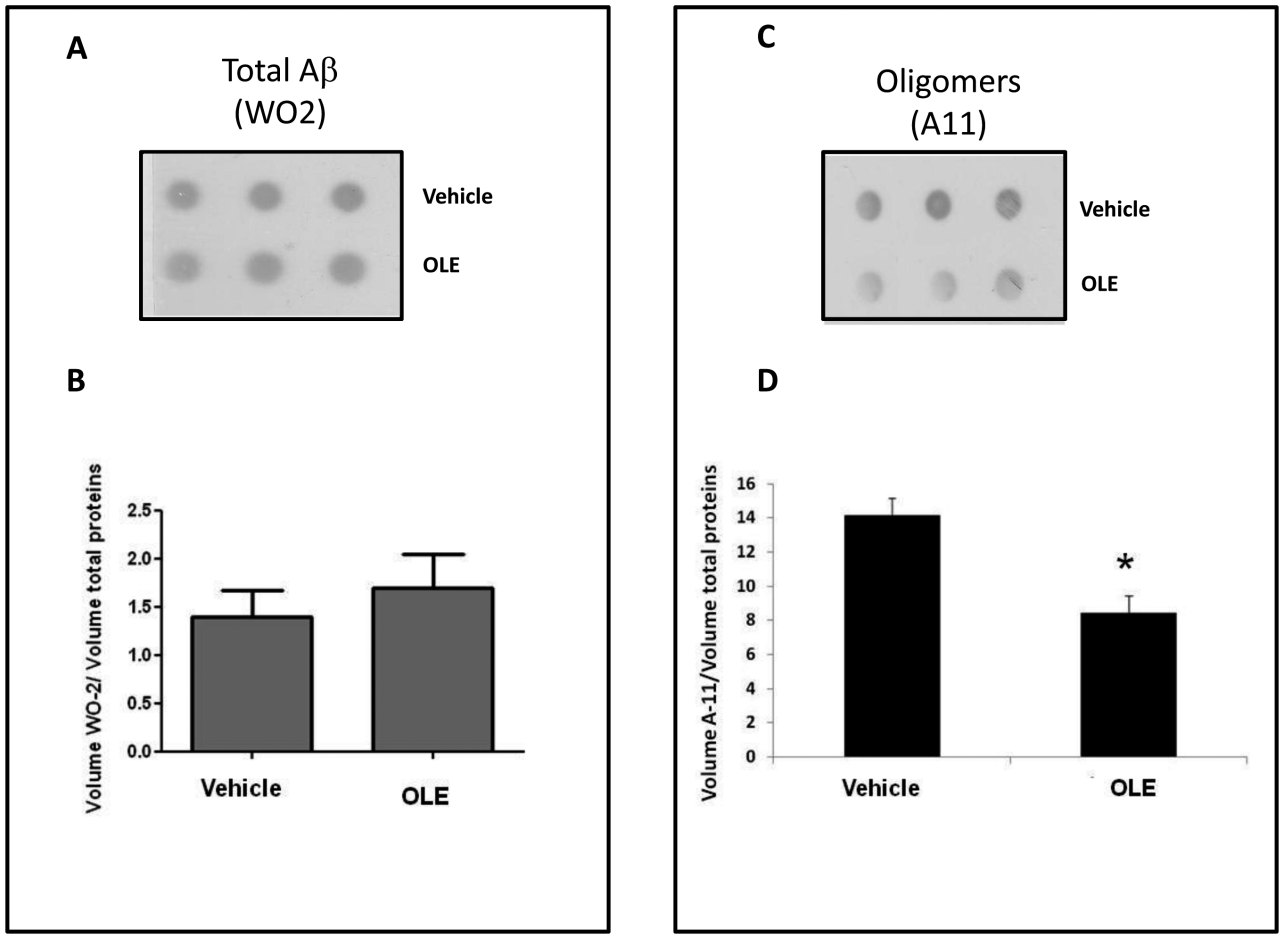
Effect of OLE on Aβ oligomer production. Representative dot blot of (**A**) total Aβ (WO2) and (**C**) Aβ oligomers (A11) in CL2006 transgenic worms fed with vehicle or with 50 µM OLE at L2 larval stage. Equal amounts of proteins from worm lysates (5 µg) were spotted in triplicate. Total proteins on the blotted membranes were stained using a 0.1% Red Ponceau solution and were used to normalize the immuno-specific signal for protein loading. The mean volume of the Aβ-reactive and Red Ponceau-dyed spots was determined using the Progenesis SameSpots software (Nonlinear Dynamics, UK). Immunoreactivity of WO2 (**B**) or A11 (**D**), from three independent experiments (n = 9), was expressed as the mean volume of the immunoreactive band/volume of Ponceau-dyed proteins±SD.*p<0.05 vs. vehicle (Student’s t-test).

Such beneficial effects of OLE were not related to its ability to modify the steady-state of Aβ by interfering with its production and/or degradation. In fact no significant difference in the Aβ immunoreactive signal was observed between worms fed with vehicle or with 50 µM OLE at L2 larval stage, as indicated by the dot blot analysis performed on CL2006 worm lysates using an antibody specific for total Aβ (WO2) (Fig. 3A, B). It is known that the expression of Aβ in paralyzed CL2006 worms is accompanied by an increase of the A11-positive oligomer production [48]. We therefore performed dot blot analysis to determine whether the protective effect of OLE could be related to its ability to counteract Aβ oligomer formation. Actually, it transpired that, in nematodes fed with 50 µM OLE at L2 larval stage, the A11-immunoreactivity was reduced by 40% compared with that found in CL2006 worms treated with vehicle (*p*<0.05, n = 9, Student’s t-test) (Fig. 3C, D).

**Figure 4:**
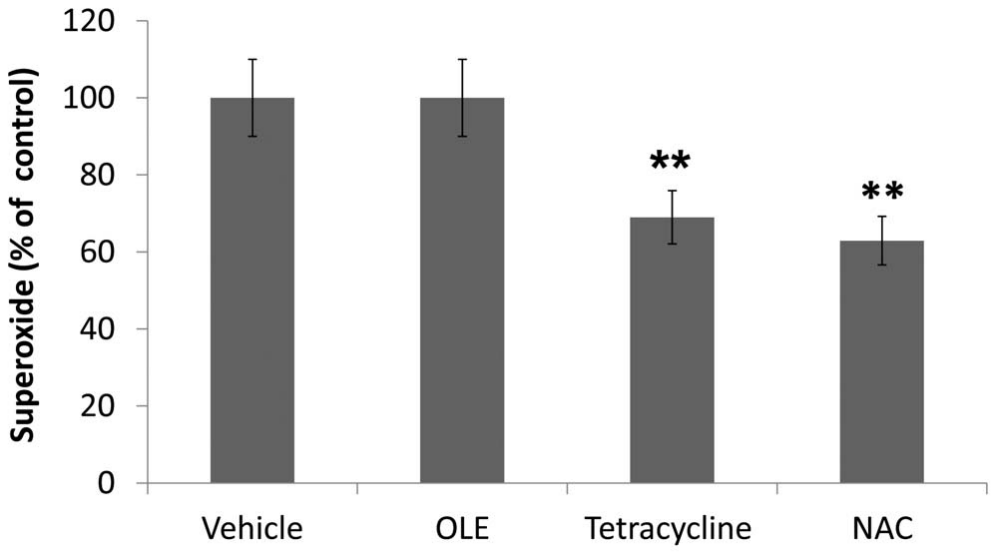
Effect of OLE on oxidative stress in CL2006 transgenic *C. elegans* strain. Egg-synchronized CL2006 transgenic worms were placed at 16 °C on *E. coli* for 48 h and then fed, at L2 larval stage, with vehicle or with 50 µM OLE for 48 h (100 µl/plate). Tetracycline and NAC were used as positive controls for antioxidant activity. Worms were then collected in 1.6 ml 1% Tween 20 in PBS and the NBT assay was done as described in Materials and Methods. Results are calculated as absorbance units/mg of protein (n = 100 worms/group, three independent experiments) and expressed as % of control, i.e. the percentage of superoxide produced by drug-treated CL2006 worms, considering 100% that produced by vehicle treated ones.**p<0.01 vs. vehicle-treated CL2006 worms, according to One-way ANOVA and Bonferroni’s post test analysis.

The increased production of superoxide precedes the paralysis in the transgenic *C. elegans* CL2006 strain constitutively expressing the human Aβ_3–42_ peptide [45], [46]; this was observed under experimental conditions (+64% of superoxide production in CL2006 worms as compared to the CL802 control strain. *p*<0.001, Student’s t-test, n = 100 worms/group). In the same strain, the protective effect of tetracyclines and of a *Ginko biloba* extract against worm paralysis was found to be associated with a reduced superoxide production [48], [49]. An *in vitro* and *in vivo* ROS-scavenging effect of chronic OLE treatment has been reported as well [51] and this is a likely explanation for its ability to reduce lipid peroxidation. We then investigated whether, besides the previously described anti-fibrillogenic effect, an antioxidant action contributed to OLE protection against Aβ toxicity. To this end, CL2006 worms were fed with vehicle or with 50 µM OLE at L2 larval stage, followed after 48 h by superoxide measurement. We found that OLE treatment did not reduce the level of intracellular superoxide, whereas a significant reduction by 37% and 31% with respect to vehicle-fed worms, was observed in nematodes treated for 24 h with antioxidant compounds such as 5.0 mM NAC or 50 µM tetracycline, respectively (Fig. 4). We conclude that the protective effect of OLE in Aβ-expressing worms is not related to its antioxidant activity. This matches the conclusion reached when we investigated OLE protection against Aβ and amylin aggregates in cultured cells [30], [40].

Overall, our data show that OLE treatment of the *C. elegans* CL2006 strain at early larval stage is significantly effective against Aβ oligomerization, fibrillization and aggregate deposition, without a significant reduction in Aβ levels. The strongly reduced extent of plaque deposition and oligomer level results in a strong protection against the pathological phenotype, such that the worms recover good motility and viability and improve their survival with respect to those fed with vehicle.

## Discussion

Interest in amyloid diseases has increased since it has become clear that this classification encompasses several pathologies. These pathologies affect different tissues and organs with various outcomes, but are all characterized by the presence of toxic protein aggregates. Such diseases typically manifest after a very long period of asymptomatic development; for this reason great attention is being paid to the identification of dietary regimens and diet components that can provide prevention or at least delay the onset and reduce the severity of the disease. In this respect, many studies have investigated the MD, with particular focus on red wine and EVOO, which were identified as important sources of polyphenolic substances with clear anti-aggregation power [25]–[34]. Our recent research on OLE, the main polyphenolic component of EVOO, corroborates such expectations. In fact, we found that OLE, once introduced in the peptide aggregation mixture, blocks the *in vitro* formation of toxic pre-fibrillar oligomers of both human amylin and Aβ_11–42_, eventually leading to the generation of harmless protofibrils and poorly structured higher order aggregates [30], [40].

However, the data on OLE from our and other laboratories came only from *in vitro* experiments using cultured cells as a biological model; accordingly, the next necessary step was to prove OLE efficacy in a living organism. We chose *C. elegans* for several reasons: in spite of its great simplicity (it is made of 959 somatic cells, it lacks a circulatory system and a central nervous system, but 302 of its cells are neurons), 60% of its genes are homologous to human ones and 12 out of its 17 signal transduction pathways are conserved in humans. It has a very short generation time (from egg to egg in 3–4 days), thus producing numerous clonal progeny, and a short lifespan that, for the CL2006 transgenic strain, hardly exceeds two weeks. In this strain the deposition of amyloid aggregates of Aβ_3–42_ in the muscles is age-dependent (as it is in man) and leads to the paralysis of the worm, a phenotype which can be easily and clearly scored.

By using this model we confirmed that OLE can protect against amyloid toxicity not only *in vitro*, but also *in vivo*. In CL2006 worms, constitutively expressing Aβ protein, the activity of OLE in preventing the amyloid-induced paralysis was specific, dose- and time-dependent (its IC_50_ was significantly lower when treatment started at L2 rather than at L3). A little protective effect was also observed on the paralysis caused in CL4176 worms by the temperature-induced Aβ aggregation, but only when OLE was administered before the induction of transgene expression.

These results agrees with those obtained *in vitro* and show that cell viability is maximally preserved when the aggregating peptide is exposed to OLE from the beginning of the aggregation process. This suggests that the main action of OLE consists in hindering the formation of toxic oligomeric species [30], probably through a direct interaction with the monomeric peptide. In turn, the significant reduction of the level of toxic oligomers relieves the animal from their toxic effects. Actually, it is increasingly recognized that oligomer toxicity results, at least in part, from their interaction with the cell membrane, with membrane alterations and disassembly resulting in derangement of the selective ion permeability [52]. Our interpretation was further confirmed by the observation that A11 immunoreactivity (given by the presence of toxic pre-fibrillar oligomers) was significantly reduced in OLE-fed CL2006 worms compared to the vehicle-treated ones. Moreover, the analysis of fibrillar deposits in the whole worm with the X-34 dye, which recognizes β-amyloid aggregates but not oligomers [50], showed the complete absence of plaques in OLE-fed animals. This was well beyond our expectations since OLE was not able to prevent the *in vitro* deposition of some pre-fibrillar material [30], [40]. The complexity of the cellular environment, much greater than that present inside a test tube, seems to improve OLE inhibitory activity, extending it beyond the inhibition of oligomer formation. By showing the absence in OLE fed worms of fibrillar deposits and the reduction of oligomeric assemblies, all with no reduction of total Aβ content, our results suggest that Aβ homeostasis is maintained by non-degradative pathways. Interestingly, a single administration of OLE is enough to achieve the maximal protective response; in fact, repeated treatments neither further inhibited worm paralysis nor prolonged their survival. In addition, we found that the dose of OLE needed to obtain the same effects as the 50 µM at L2 was increased by ten times at L3 (Fig. 1D). It is proposed that the dose of OLE needed to maintain reduced oligomer levels and worm viability increases with age as far as the tendency of the Aβ peptide to aggregate, due to the presence of previously-formed tiny aggregates or seeds. A possible explanation of the fact that a further administration of OLE after that at L2 is redundant could be that OLE remains in the cells for prolonged periods, even when the worms are transferred to new culture plates that do not contain the drug, making any further OLE implementation ineffective. Our survival data agree with this view. In fact, CL2006 survival was significantly increased after a single OLE administration at L2, whereas a second administration, 8 days later (i.e. in the middle of the lifespan), did not further improve the result.

OLE, like all polyphenols, has antioxidant properties, and reactive oxygen species production is a typical outcome of amyloid deposition. Other molecules that were shown to be effective as inhibitors of amyloid deposition and paralysis in CL2006 strain, like tetracyclines, also induced a reduction of superoxide production [48]. However, this anti-oxidant effect was not seen in OLE-fed worms. This result confirms our idea that the beneficial effect of OLE cannot be traced back to its antioxidant properties. In fact, if cultured cells are concomitantly exposed to toxic amyloid aggregates and to OLE, their viability is not recovered. Actually the protection occurs only when cells are exposed to aggregates grown in the presence of OLE [30], [40], thus devoid of toxic oligomeric species. The results obtained in this invertebrate model confirm that, as show for other polyphenols, a key aspect of OLE protection against amyloid toxicity results from its anti-aggregation properties [25], [29], [53], [54]. Notice that, in these transgenic *C. elegans* strains, the oxidative stress performs a minor role in amyloid toxicity. In fact L-ascorbic acid treatment, which reduces superoxide levels, has no protective effect on the paralysis phenotype [43], which is mainly determined by Aβ oligomerization.

## Conclusions

OLE, the main polyphenol of EVOO, is capable of interfering with the toxic amyloid aggregation of Aβ not only *in vitro*, but also *in vivo* in an invertebrate model of AD and sIBM. OLE-fed worms showed a reduced paralysis and a prolonged lifespan. Though further research is needed to clarify the exact molecular and cellular mechanisms underlying OLE protective activity, our results suggest that, at least in our experimental model, OLE does not alter the rate of production and degradation of Aβ nor acts as an antioxidant; rather, it seems to directly interfere with the aggregation process inhibiting the formation of soluble toxic oligomers and amyloid fibrils, as has already been observed *in vitro*.

## Supporting Information

Figure S1 **Effect of OLE on Aβ-induced paralysis in CL4176 transgenic** ***C. elegans*** **strain.** (**A**) Diagram illustrating the paralysis assay performed in CL4176 worms, showing when the OLE was administered, when the temperature was increased and when the paralysis was determined. (**B**) Percentage of paralyzed worms fed with 50–100 µM OLE 30 h before the temperature increase (corresponding to L1 larval stage). Egg-synchronized CL4176 worms were placed at 16 °C on fresh NMG plates seeded with OP50 *E. coli* and, at L1 were fed with vehicle or OLE (50–100 µM). Tetracycline at 50 µM, was administered at L3 as positive control. The number of paralyzed worms was scored 42 h after temperature induction. Data are shown as percentage±SD of paralyzed worms to vehicle treated ones (n = 100 worms/group, 3 independent assays). *p<0.05 and **p<0.01 vs. CL4176 worms fed with vehicle (One-way ANOVA test).
(TIF)
