# Supplementary figures and images for: Oleuropein Aglycone Protects Transgenic C. elegans Strains Expressing Aβ42 by Reducing Plaque Load and Motor Deficit
Source: PLoS One. 2013 Mar 8;8(3):e58893. doi: 10.1371/journal.pone.0058893 (PMC3592812; doi:10.1371/journal.pone.0058893)

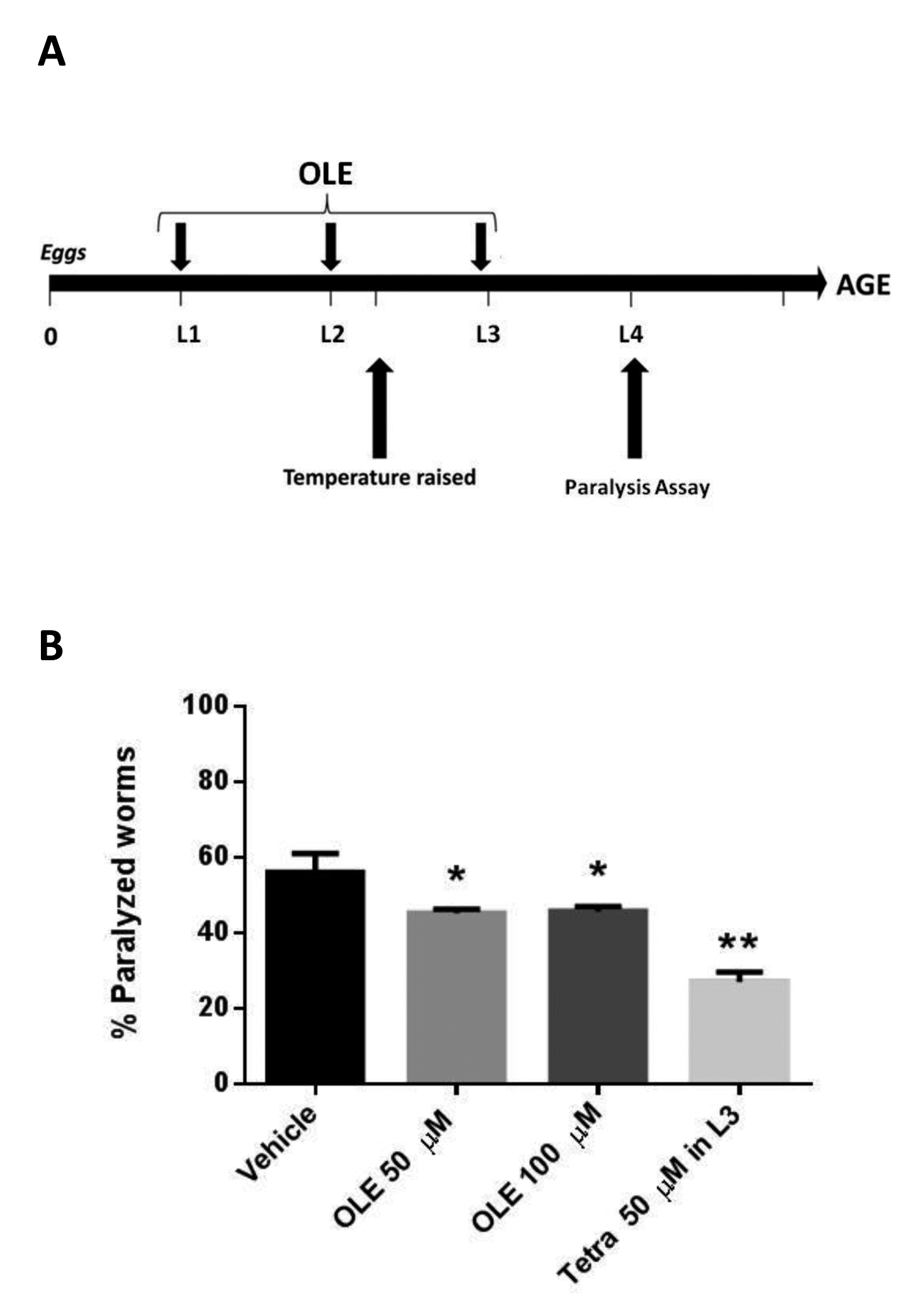

Supplement: Figure S1 — Effect of OLE on Aβ-induced paralysis in CL4176 transgenic C. elegans strain. (A) Diagram illustrating the paralysis assay performed in CL4176 worms, showing when the OLE was administered, when the temperature was increased and when the paralysis was determined. (B) Percentage of paralyzed worms fed with 50–100 µM OLE 30 h before the temperature increase (corresponding to L1 larval stage). Egg-synchronized CL4176 worms were placed at 16 °C on fresh NMG plates seeded with OP50 E. coli and, at L1 were fed with vehicle or OLE (50–100 µM). Tetracycline at 50 µM, was administered at L3 as positive control. The number of paralyzed worms was scored 42 h after temperature induction. Data are shown as percentage±SD of paralyzed worms to vehicle treated ones (n = 100 worms/group, 3 independent assays). *p<0.05 and **p<0.01 vs. CL4176 worms fed with vehicle (One-way ANOVA test). (TIF) [file pone.0058893.s001.tif]
